# Profiling of H3K4me3 and H3K27me3 and Their Roles in Gene Subfunctionalization in Allotetraploid Cotton

**DOI:** 10.3389/fpls.2021.761059

**Published:** 2021-12-15

**Authors:** Aicen Zhang, Yangyang Wei, Yining Shi, Xiaojuan Deng, Jingjing Gao, Yilong Feng, Dongyang Zheng, Xuejiao Cheng, Zhaoguo Li, Tao Wang, Kunbo Wang, Fang Liu, Renhai Peng, Wenli Zhang

**Affiliations:** ^1^State Key Laboratory for Crop Genetics and Germplasm Enhancement, JCIC-MCP, CIC-MCP, Nanjing Agricultural University, Nanjing, China; ^2^Biological and Food Engineering, Anyang Institute of Technology, Anyang, China; ^3^College of Agronomy, Xinjiang Agricultural University, Ürümqi, China; ^4^Zhengzhou Research Base, State Key Laboratory of Cotton Biology, Zhengzhou University/Institute of Cotton Research, Chinese Academy of Agricultural Sciences, Anyang, China

**Keywords:** H3K4me3, H3K27me3, ChIP-seq, gene expression, subfunctionalization, regulatory network, cotton

## Abstract

Cotton is an excellent model for studying crop polyploidization and domestication. Chromatin profiling helps to reveal how histone modifications are involved in controlling differential gene expression between A and D subgenomes in allotetraploid cotton. However, the detailed profiling and functional characterization of broad H3K4me3 and H3K27me3 are still understudied in cotton. In this study, we conducted H3K4me3- and H3K27me3-related ChIP-seq followed by comprehensively characterizing their roles in regulating gene transcription in cotton. We found that H3K4me3 and H3K27me3 exhibited active and repressive roles in regulating the expression of genes between A and D subgenomes, respectively. More importantly, H3K4me3 exhibited enrichment level-, position-, and distance-related impacts on expression levels of related genes. Distinct GO term enrichment occurred between A/D-specific and homeologous genes with broad H3K4me3 enrichment in promoters and gene bodies, suggesting that broad H3K4me3-marked genes might have some unique biological functions between A and D subgenome. An anticorrelation between H3K27me3 enrichment and expression levels of homeologous genes was more pronounced in the A subgenome relative to the D subgenome, reflecting distinct enrichment of H3K27me3 in homeologous genes between A and D subgenome. In addition, H3K4me3 and H3K27me3 marks can indirectly influence gene expression through regulatory networks with TF mediation. Thus, our study provides detailed insights into functions of H3K4me3 and H3K27me3 in regulating differential gene expression and subfunctionalization of homeologous genes, therefore serving as a driving force for polyploidization and domestication in cotton.

## Introduction

The core nucleosome is the basic unit of chromatin. It comprises a core histone octamer, consisting of two copies of each histone H3, H4, H2A, and H2B, wrapped by approximately 147 bp of double-helix DNA (Kornberg, 1974). The exposed N-terminal tails of core histones, especially for histone H3, are subjected to various covalent modifications like methylation and acetylation with distinct biological consequences (Fuchs et al., 2006; Kouzarides, 2007; Bannister and Kouzarides, 2011). In plants, individual histone modifications, especially combinatorial actions of multiple modifications, play essential roles during entire processes of normal growth and development (Berr et al., 2011; Deal and Henikoff, 2011; Boycheva et al., 2014; Han et al., 2019) and in response to diverse environmental cues (Kim J. M. et al., 2015; Ramirez-Prado et al., 2018; Alonso et al., 2019; Ueda and Seki, 2020). In particular, aberrant histone modifications frequently cause severe developmental defects in plants (Tian and Chen, 2001; Sanders et al., 2017; Deevy and Bracken, 2019), indicating histone modifications are indispensable for normal plant growth and development.

Histone methylation exhibits lysine site- and methylation degree-dependent effects on chromatin structure and gene transcription. Selective lysine residues can be mono-, di-, and trimethylated with distinct biological outcomes (Sims et al., 2003; Tariq and Paszkowski, 2004; Zhang et al., 2009; Liu et al., 2010; Stillman, 2018). H3K4me3 is a major active histone mark in eukaryotes (Santos-Rosa et al., 2002; Zhang et al., 2015a). By contrast, H3K27me3 is a repressive mark with significant biological relevance in eukaryotes, including plants (Zheng and Chen, 2011; Wiles and Selker, 2017).

So far, H3K4me3 and H3K27me3 are well-studied marks in plants (Deal and Henikoff, 2011). They play crucial roles in regulating gene expression during normal growth and development and responses to environmental cues in plants (Probst and Mittelsten Scheid, 2015; Chen D. H. et al., 2018). In general, H3K4me3 primarily distributes around the TSS or gene bodies of expressed genes, which is directly related to gene activation (Wang et al., 2009; Zhang et al., 2009; He et al., 2010). In contrast, H3K27me3 is primarily enriched in the promoters and gene bodies of repressed genes responsible for silencing conditionally expressed genes (Zhang et al., 2007; Wang et al., 2009; Deal and Henikoff, 2010; He et al., 2010). Moreover, the H3K27me3 mark has been recently found to be more associated with homeologs less expressed in polyploidy wheat (Ramirez-Gonzalez et al., 2018), indicating involvement of this mark in the expression bias of homeologs in the polyploidy genome.

Cotton (*Gossypium* spp.) is one of the major suppliers of natural textile fibers and oilseeds around the world. Allotetraploid cotton is an excellent model system for studying crop polyploidization and domestication. The availability of high-quality genome sequences for cultivated allotetraploid species and their wild relatives (Wang et al., 2012, 2019; Li F. et al., 2015; Zhang et al., 2015b; Chen et al., 2020) has promoted evolutionary and functional genomics studies (Zaidi et al., 2018), therefore, deepening our understanding of cotton biology and benefiting yield and fiber improvement in cotton breeding. However, when compared with tremendous progress in other model plants, like *Arabidopsis*, rice, and maize, histone modification-related epigenomic studies are largely understudied in cotton (Wang et al., 2016, 2017; Zheng et al., 2016; You et al., 2017; Tao et al., 2020), especially H3K27me3, a repressive mark for regulating genes involved in development and stress responses.

Here, we conducted H3K4me3 and H3K27me3 ChIP-seq assays using leaf tissue from the allotetraploid cotton cultivar *Gossypium hirsutum* (TM-1). We examined subgenomic distribution of both marks. In particular, we comprehensively investigated the roles of H3K4me3 and H3K27me3 marks in regulating the differential expression of genes and homeologous genes between the A and D subgenomes.

## Materials and Methods

### Plant Material and Growth Conditions

The allotetraploid cotton cultivar *G. hirsutum* was used in this study. Cotton seeds were soaked in water for 24 h, then transferred to soil in pots and continued to grow in a greenhouse with 60% humidity at 28°C/25°C and 16/8-h light/dark cycle. Two and three true leaves collected from 20-day-old plants were ground into a fine powder using liquid nitrogen. The ground powder can be used immediately or kept at −80°C for later use.

### RNA-Seq

Total RNA was extracted from ground powder using TRIzol (Thermo Fisher Scientific). Total RNA was treated with DNaseI to completely remove contamination of genomic DNA. mRNA was enriched from DNaseI-treated RNA for library preparation and sequenced on Illumina HiSeq4000.

### ChIP-Seq Assay

Twenty-day-old cotton leaves were used for nuclei preparation. The nuclei were extracted using a nuclei isolation buffer (NIB, pH 5.0, 1.0 M glucose, 0.1 M citric acid, 80 mM KCl, 10 mM EDTA, 1% Triton X-100 prepared fresh just before use). The nuclei were purified using 1 × nuclei washing buffer (NWB, pH 5.0, 1 M glucose, 0.1 M Na-citrate, 1%Triton X-100 prepared fresh just before use). The purified nuclei were resuspended using 600 μl MNB buffer (50%, w/v sucrose, 50 mM Tris–HCl, pH 7.5, 4 mM MgCl_2_, 1 mM CaCl_2_) for MNase digestion at 37°C for 10 min. A digestion mix was pelleted at 13,000 rpm for 10 min at 4°C and the supernatant was transferred into a new 1.5 ml tube. The digested nuclei pellet was resuspended using lysis buffer (1 mM Tris–HCl, pH 7.5, 0.1 mM PMSF, 2%, v/v Complete Mini) and left on ice for 1 h. After centrifugation, the supernatant was transferred into the 1.5 ml tube containing digested chromatin. ChIP incubation buffer was added to the digested chromatin to make a total of 1.7 ml. The remaining steps were conducted following the published procedures (Zhang et al., 2012), namely, antibody incubation followed by adding protein A-sepharose beads, bead washing, elution, and purification of ChIPed DNA for library preparation. The prepared libraries were finally sequenced on the Illumina platform (Illumina HiSeq4000).

### Processing of Sequencing Data

Raw reads of all sequencing data were trimmed using fastp (Chen S. et al., 2018) based on the quality value (*Q* ≥ 25) and read length (≥20 bp). The trimmed reads from RNA-seq and ChIP-seq were mapped to the *G. hirsutum* reference genome (Zhang et al., 2015b) with Hisat2 (Kim D. et al., 2015) and Bowtie2 aligner (Langmead and Salzberg, 2012), respectively. The remaining RNA-seq data of different tissues were obtained from the previously published data (Zhang et al., 2015b), and processed according to the procedures described above. For ChIP-seq data, any PCR duplicates were removed using Picard. Aligned reads with mapping quality (MapQ) less than 30 were removed using samtools (Li et al., 2009). The bam files were converted to bigwig file and normalized by RPKM (Reads Per Kilobase per Million mapped reads) using deeptools (Ramirez et al., 2016), then Integrative Genomics Viewer (IGV) (Thorvaldsdottir et al., 2013) was used to visualize read distribution across the genome.

### Identification of Differentially Expressed Homeologous Genes

Fragments Per Kilobase of exon model per Million mapped fragments (FPKM) computed from StringTie (Pertea et al., 2015) was used to measure the expression level of each gene. Gene annotations were obtained from CottonGen (Yu et al., 2014). Only genes with FPKM ≥ 1 were considered as expressed ones. Homeologous gene pairs with FPKM ≥ 1 in at least one of the subgenomes were used for further analyses. The homeologous gene pairs (Supplementary Table 1) were identified using reciprocal BLAST hits between A and D homeologs as previously reported (Zhang et al., 2015b). Differentially expressed homeologous genes were analyzed using the limma R package. The *P* values were adjusted using the BH method at α = 0.05 (Benjamini and Hochberg, 1995). Corrected *P* values of 0.05 and log_2_ (fold-change) values of 1 were set as the threshold for assessing differential expression levels.

### ChIP-Seq Data Analyses

In this study, we only used rep I data of H3K4me3 and H3K27me3 for peak calling and subsequent analyses, and we used rep II data to validate the reproducibility of peaks for each mark. H3K27me3 peaks were called with “–broad” parameter on (-f BAM -g 2.3e+9 –nomodel -q 0.01 –broad –broad-cutoff 0.1) and H3K4me3 peaks were called with off (-f BAM -g 2.3e+9 –nomodel -q 0.01) using the MACS2 software (Zhang et al., 2008). The ChIPpeakAnno package (Zhu et al., 2010) was used for peak annotation. Bedtools (Quinlan and Hall, 2010) were used to correlate peaks with genome loci, genes (including 1,000 bp region upstream of the TSS and 1,000 bp region downstream of the TTS) overlapping H3K4me3 or H3K27me3 peaks were considered as peak-related genes. A custom script was used to calculate normalized read counts of related genes.

### Regulatory Network Analyses

The biased genes between the A and D subgenomes were chosen to construct gene co-expression networks using the R package WGCNA (Langfelder and Horvath, 2008). The blockwiseModules function was used for network construction with the following parameters: power = 16, minModuleSize = 30, mergeCutHeight = 0.25, corType = “pearson.” The same expression matrix used for WGCNA analyses was used for regulatory network analyses with the R package GENIE3 (Huynh-Thu et al., 2010). All TF annotations were obtained from PlnTFDB version 3.0 (Jin et al., 2014) and CottonFGD^1^.

### Gene Ontology Enrichment Analyses

Gene Ontology enrichment analyses were conducted using agriGO v2.0 (Tian et al., 2017). GO terms with an FDR less than 0.05 were considered as being significantly enriched.

### Bimolecular Fluorescent Complimentary (BiFC) Assay

To verify protein–protein interactions among PRE6, Rf2b, and bHLH63 as shown in Figure 4B, we performed transient transformation related Bimolecular Fluorescent Complimentary (BiFC) assays using *Nicotiana benthamiana*. To generate vectors of pXY104 with the expression cassette of 35S: *PRE6-cYFP*, R*f2b-cYFP* and *bHLH63-cYFP*, we fused the coding sequences of PRE6, Rf2b, and bHLH63 to the C-terminal half of YFP. Meanwhile, we tagged each gene with the N-terminal half of YFP to generate vectors of pXY104 with the expression cassette of 35S:*PRE6-nYFP*, *Rf2b-nYFP* and *bHLH63-nYFP* (Supplementary Table 2). All plasmids were individually transferred into *Agrobacterium tumefactions* (GV3101), and cultured in LB medium at 28°C until OD600 reached 1.2–1.5. After collecting the strains by centrifugation at 5,000 rpm at 4°C, the strains were individually suspended in injection buffer (10 mM MES-KOH, 10 mM MgCl_2_, 40 μM AS, pH = 5.7). After incubation at room temperature for 2 h, the bacteria containing cYFP/nYFP, Rf2b-cYFP/Rf2b-nYFP, Rf2b-cYFP/PRE6-nYFP, Rf2b-cYFP/bHLH63-nYFP, PRE6-cYFP/PRE6-nYFP, PRE6-cYFP/bHLH63-nYFP, and bHLH63-cYFP/bHLH63-nYFP were combined in 1:1 ratio, and were then individually co-injected into young and healthy *N. benthamiana* leaves for transient expression. The fluorescent signal was monitored and recorded using the Leica DMi8 confocal laser scanning microscope at 72 h post-infiltration, at least three images per injection. The functional validation for each combination was repeated by at least three separate injections.

**FIGURE 1:**
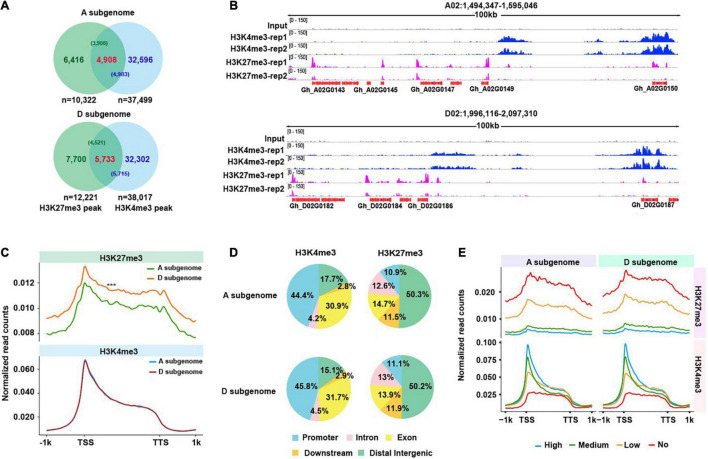
Distribution of H3K4me3 and H3K27me3 marks. **(A)** Venn plots illustrating overlaps of H3K4me3 and H3K27me3 peaks, and H3K4me3/H3K27me3 bivalents identified from A and D subgenome, respectively. The number in each bracket represents the number of H3K4me3 (or H3K27me3) peaks that overlap with H3K27me3 (or H3K4me3). **(B)** Representative IGV snapshots across a 100 kb window from chromosome A02 and chromosome D02 show the enrichment of H3K4me3 and H3K27me3 peaks in the A and D subgenomes. **(C)** Curve plots show the profile of normalized H3K4me3 and H3K27me3 read counts from 1 kb upstream of the TSSs to 1 kb downstream of the TTSs across all genes from two subgenomes. A significance test was determined using the Wilcoxon rank-sum test, ***p < 0.001. **(D)** Distribution of H3K4me3 and H3K27me3 in different functional sub-genomic annotations, namely, promoters (upstream 2 kb), exons, introns, downstream (2 kb) and distal intergenic regions. **(E)** Curve plots show the profile of normalized H3K4me3 and H3K27me3 read counts from 1 kb upstream of the TSS to 1 kb downstream of the TTS of all genes from the A and D subgenomes. All genes were divided into four subtypes according to their FPKM values (high, medium, low, and no expression).

**FIGURE 2:**
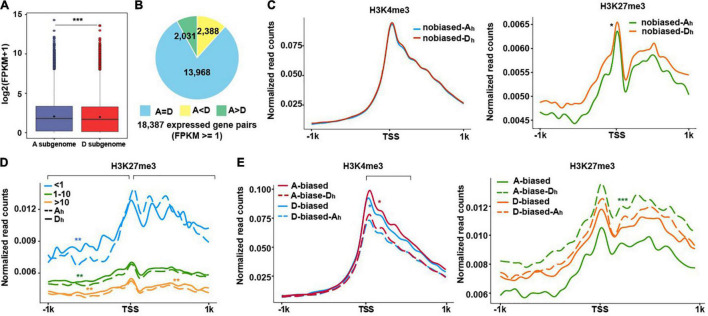
Subgenomic variations in relationships between H3K4me3/H3K27me3 and gene expression levels. **(A)** The boxplot show the average gene expression levels from A and D subgenome. A significance test was determined using the Wilcoxon rank-sum test, ***p < 0.001. **(B)** Pie charts show the number of homeologs with equal (A = D), A homeolog-biased (A > D), and D homeolog-biased (A < D) expression. **(C–E)** The profiles of normalized H3K4me3 and H3K27me3 read counts from ± 1 kb around TSS of the homeologous genes from two subgenomes. H3K4me3 (left) and H3K27me3 (right) normalized read counts for no biased gene pairs **(C)**. H3K27me3 normalized read counts for no biased genes that were divided into three groups (FPKM < 1, 1–10, and ≥10) **(D)**. H3K4me3 (left) and H3K27me3 (right) normalized read counts for biased gene pairs **(E)**. The significance test was determined using the Wilcoxon rank-sum test, *p < 0.05, **p < 0.01, ***p < 0.001.

**FIGURE 3:**
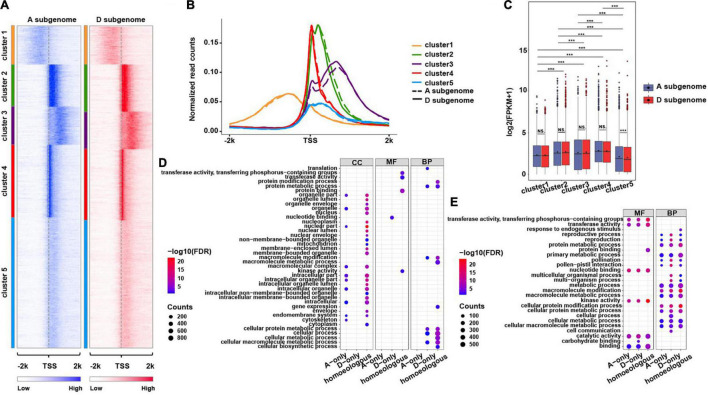
Distribution of H3K4me3 mark around TSSs of the genes in two subgenomes. **(A)** Heatmaps showing five *k*-means clusters for H3K4me3 distributed around ± 2 kb of TSSs of the overlapping genes in A and D subgenome. The color represents H3K4me3 enrichment levels. **(B)** The profiles of normalized H3K4me3 read counts around ± 2 kb of TSSs of the genes from each cluster characterized in **(A)**. **(C)** The boxplots show expression levels of the genes from each cluster in two subgenomes. A significance test was determined using the Wilcoxon rank-sum test, ***p < 0.001. **(D,E)** Functional GO term enrichment analyses of the genes from Cluster1 **(D)** and Cluster3 **(E)**, the related genes were divided into three types, A subgenome-only, homeologous (homeologous gene pairs in the same cluster in A and D subgenome) and D subgenome-only, the size of each dot represents the number of genes, and the color key indicates – log10 (FDR).

**FIGURE 4:**
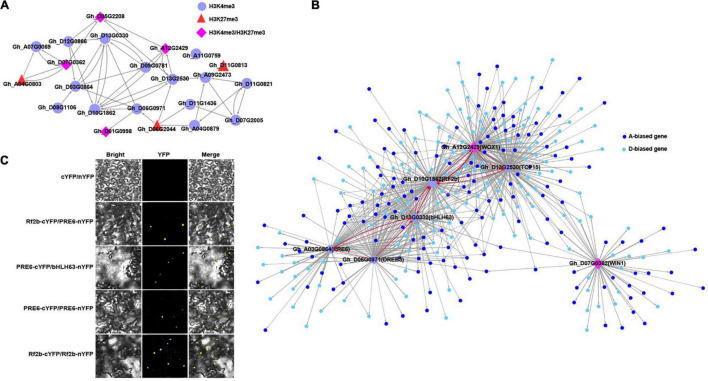
Regulatory network in cotton leaf tissue. **(A)** Overview of predicted regulatory network containing 22 hub TFs with different marks in the blue module. The TFs with H3K4me3, H3K27me3, and H3K4me3/H3K27me3 marks were indicated using different shapes and colors; the arrow represents the direction of regulation. **(B)** The directional regulatory network show the top 7 TFs with the most edges in **(A)** and their predicted regulated genes (small nodes with different colors), the arrow represents the direction of regulation, the red line indicates interactions between TFs, thickness of the line represents the weight value. **(C)** BiFC analyses of protein–protein interactions of PRE6, Rf2b and bHLH63. Bright, YFP, and Merge (bright-field and yellow fluorescent), Rf2b-cYFP/PRE6-nYFP: the C-terminal half of YFP was fused to the C-terminal of PRE6, Rf2b and bHLH63 to generate PRE6-cYFP, Rf2b-cYFP, and Bhlh63-cYFP, whereas the N-terminal half of YFP was fused to the N-terminal of PRE6, Rf2b, and bHLH63 to generate PRE6-nYFP, Rf2b-cYFP, and bHLH63-cYFP; *Agrobacterium* combination of Rf2b-cYFP/PRE6-nYFP, PRE6-cYFP/bHLH63-nYFP, PRE6-cYFP/PRE6-nYFP, and Rf2b-cYFP/Rf2b-nYFP were individually co-injected into *N. benthamiana* leaves for transient expression. The fluorescent signal was monitored and recorded by confocal microscopy at 72 h post-infiltration. Bar: 25 μm.

## Results

### Global Distribution of H3K4me3 and H3K27me3 in Cotton

To examine global distributions of H3K4me3 and H3K27me3 in the allotetraploid cotton, we conducted each mark-related ChIP-seq (Supplementary Table 3). We found that biological replicates of each mark were well correlated (Supplementary Figure 1). After data processing and peak calling, we identified total 75,516 H3K4me3 peaks (37,499 peaks in A subgenome and 38,017 peaks in D subgenome), 22,543 H3K27me3 peaks (10,322 peaks in A subgenome and 12,221 peaks in D subgenome) and 10,641 loci with at least 1bp overlap between H3K4me3 and H3K27me3 peaks (4,908 loci in A subgenome and 5,733 loci in D subgenome) (Figures 1A,B). The representative IGV snapshots (Figure 1B) show reproducible distribution of each mark in the A and D subgenomes. We then plotted normalized H3K4me3 and H3K27me3 ChIP-seq read counts across ± 1kb of the TSS and the TTS of all genes in the A and D subgenomes. We observed a distinct genic distribution of each mark, H3K4me3 was highly enriched immediately downstream of the TSS and extended to the whole gene body, by contrast, H3K27me3 primarily covered the whole gene body (Figure 1C). Strikingly, we found that distributions of normalized read counts for H3K4me3 were similar between A and D subgenome, whereas distributions of normalized read counts for H3K27me3 were higher in D subgenome relative to A subgenome.

To visualize the distribution of each mark in the A and D subgenomes, we partitioned each subgenome into five functionally annotated subregions, namely, promoters, exons, introns, downstream of TTS, and distal intergenic regions. We observed subtle differences in H3K4me3 but similar distributions for H3K27me3 between A and the D subgenomes (Figure 1D). Compared to the D subgenome, A subgenome had approximately 2% more H3K4me3 distributed in distal intergenic regions, and 1.4% less H3K4me3 distributed in promoters. Moreover, a distinct subgenomic distribution was observed for each mark, H3K4me3 exhibited the highest distribution in promoters, while H3K27me3 had the highest distribution in distal intergenic regions, suggesting a potential mark-dependent functional divergence. To assess an association between H3K4me3 or H3K27me3 enrichment levels and gene expression levels in the A and D subgenome, we classified all genes in the A and D subgenome into four subtypes (high, medium, low, and no expression) according to FPKM values. We then plotted normalized H3K4me3 or H3K27me3 read counts around ± 1kb of the TSS and the TTS of genes. We found that H3K4me3 enrichment levels, indicative of normalized read counts, exhibited a positive correlation with gene expression levels, whereas H3K27me3 enrichment levels were anti-correlated with gene expression levels in A and D subgenome (Figure 1E). Consistent with previous reports in other plant species, H3K4me3 and H3K27me3 also have contrasting roles in regulating gene expression in cotton, the former can facilitate gene expression whereas the latter usually suppresses gene expression.

### Distinct Roles of H3K4me3 and H3K27me3 in Regulating Expression of Homeologous Genes

After comparing expression levels of genes between the A and D subgenomes, we observed that genes in A subgenome generally expressed more than those in D subgenome (Figure 2A), indicating subgenome-biased expression in cotton. To examine how H3K4me3 and H3K27me3 are involved in regulating the differential expression of homeologous genes, we classified 18,387 expressed homeologous gene pairs into three subtypes: non-biased expression, A = D (*n* = 13,968), representing equal expression levels between A and D; A-biased expression, A > D (*n* = 2,031), representing genes expressed more in A homeologs and D-biased expression, D > A (*n* = 2,388), representing genes expressed more in D homeologs (Figure 2B; Supplementary Figure 2; Supplementary Table 4). After comparing normalized read counts, we found that there was no difference for H3K4me3 for no biased A_h_ and D_h_ (A_h_ and D_h_ represent A homeologs and D homeologs, respectively), whereas more H3K27me3 occurred in no biased-D_h_ than no biased-A_h_, the counterparts in the A subgenome (Figure 2C).

To assess if read density changes of H3K27me3 in no biased homeologous genes are possibly related to gene expression levels, we classified no biased homeologous genes into three subtypes: FPKM < 1, 1–10, and > 10 and conducted similar plotting assays as Figure 2C. When compared with genes in the D subgenome, we observed that non-expressed genes (FPKM < 1) in A exhibited less H3K27me3 reads distributed at the upstream of TSSs, but more H3K27me3 reads distributed at the downstream of TSSs. For expressed genes (FPKM ≥ 1), H3K27me3 reads tended to have more in the D subgenome than the A subgenome across all regions examined (Figure 2D).

We conducted similar plotting analyses for A- and D-biased expressed genes. As shown in Figure 2E (left), H3K4me3 exhibited higher enrichment in A-biased and D-biased genes compared to their respective counterpart of homeologous genes (A-biased-D_h_ and D-biased-A_h_), indicating that H3K4me3 enrichment levels are directly correlated with gene expression levels. By contrast, H3K27me3 was less enriched in A-biased and D-biased genes compared to the corresponding A-biased-D_h_ and D-biased-A_h_, respectively (Figure 2E right), exhibiting an anticorrelation between H3K27me3 enrichment levels and gene expression levels. After a careful examination, we found that the difference in H3K27me3 between A-biased and A-biased-D_h_ was more pronounced than that between D-biased and D-biased-A_h_. These results showed that an anti-correlation between H3K27me3 enrichment levels and expression levels of homeologous genes was more pronounced in the A subgenome relative to the D subgenome, reflecting distinct enrichment of H3K27me3 in homeologous genes between A and D subgenome. Similarly, the roles of H3K27me3 in regulating biased expression of homeologs have been investigated in polyploidy wheat (Ramirez-Gonzalez et al., 2018), indicating potential common roles of H3K27me3 in regulating differential expression of homeologs in polyploidy plants.

### Biological Implications of Genes With Broad H3K4me3

It has been reported that genes with broad H3K4me3 enrichment have some particular biological implications, such as determination of cell identity, regulation of expression of cell-type-specific tumor suppressors, regulation of expression of genes responsible for gamete development, and pre-implantation in mammalians (Benayoun et al., 2014; Chen et al., 2015; Dahl et al., 2016; Liu et al., 2016; Lv and Chen, 2016; Zhang et al., 2016) and potential roles in photosynthesis in *Arabidopsis* (Brusslan et al., 2015). To interrogate if genes marked with broad H3K4me3 enrichment have any distinct biological implications in cotton, we conducted *k*-means clustering assay using H3K4me3 associated genes in the A and D subgenomes. We obtained five clusters of genes with distinct H3K4me3 ChIP-seq read distribution (Figures 3A,B). There were two types of genes (Cluster 1 and 3) with broad H3K4me3 enrichment in promoter and gene body regions, respectively. After comparing gene expression levels and gene length in each cluster, genes in each cluster between the A and D subgenomes had overall similar mean expression levels (Figure 3C) and mean gene length (Supplementary Figure 3A). We found that genes in Cluster 4 had the highest mean expression levels and gene length, whereas genes in Cluster 5 had the highest gene length but the lowest mean expression levels in both subgenomes and genes in Cluster 3 had the shortest gene length (Figure 3C; Supplementary Figure 3A and Supplementary Table 5). These results suggest that the impacts of H3K4me3 on gene expression may depend on its enrichment levels, position, and distance from H3K4me3 to the TSS.

We then conducted GO term enrichment analyses using genes in Clusters 1 and 3 in A and D subgenome. For the genes in Cluster 1, we found that majority of GO terms were common between A and D subgenome despite several distinct GO terms occurred between A and D subgenome. For instance, the genes in D subgenome were more enriched in macromolecular complex/metabolic processes, cellular processes and transducer activities while the genes in A subgenome were more enriched in protein binding (Supplementary Figure 4). We further divided the genes in Cluster 1 into A or D subgenome-specific and homeologous gene pairs (Supplementary Figure 3B) for re-conducting GO term enrichment assays. We observed distinct GO terms occurred between A- and D-specific genes (Figure 3D). A subgenome-only genes were more enriched in cellular component category but less enriched in molecular function and biological process categories compared to D subgenome-only genes. Homeologous gene pairs were more involved in the cellular component category but less in the molecular function category.

As illustrated in Figure 3A (heatmap) and Supplementary Figure 3A (boxplot), the genes in Cluster 3 contain broad H3K4me3 mark covering downstream 2 kb of the TSSs, but have the shortest gene length. To specifically look into biological relevance of genes with broad H3K4me3 enriched in gene body instead of extending to downstream of the genes with length less than 2 kb, we conducted similar GO term analyses using the genes in Cluster 3 with length greater than 2 kb in A and D subgenome (Supplementary Figures 5A,B). We observed subtle differences in GO terms between A and D genome. Compared to the genes in A subgenome, the genes in D subgenome were more enriched in signal transducer activity, carbohydrate binding, cell communication and pollination and pollen-pistil interaction (Supplementary Figure 5C). After dividing the genes specific for A or D subgenome and homeologous gene pairs (Supplementary Figure 5D), we found the genes in D subgenome only were more enriched in functions associated with carbohydrate binding, metabolic processes, pollination, and pollen–pistil interaction as compared to the genes in A subgenome only (Figure 3E). To test if genes with broad H3K4me3 mark exhibit tissue-specific differential expression, we conducted *k*-means clustering analyses using RNA-seq data derived from 12 distinct tissues (the public data), we found that the genes with broad H3K4me3 enrichment exhibited tissue-specific expression profiles, including significantly highly expressed genes in stamen and petal (Supplementary Figures 6A,B), suggesting that broad H3K4me3-marked genes may function in reproductive stage for flower development. These analyses suggest that broad H3K4me3-marked genes might have some unique biological functions between A and D subgenome.

### Involvement of H3K4me3 and H3K27me3 in Regulating Gene Expression Through TF-Mediated Regulatory Network

After specifically examining TFs with subgenome-related differential expression, we detected 180 and 204 TFs with biased expression in A and D subgenome, respectively (Supplementary Figure 7 and Supplementary Table 6). A- and D-biased TFs associated with H3K4me3-only, H3K27me3-only, and H3K4me3/H3K37me3 mark were summarized in Supplementary Tables 6, 7. To interrogate if H3K4me3 and H3K27me3 function in regulating gene expression through TF-mediated regulatory networks, we conducted a co-expression assay with the WGCNA R package, an expression matrix of biased genes across 12 tissues was used for the downstream analyses. We obtained 21 co-expression modules (Supplementary Figure 8A). Genes in the blue module exhibited a high association in the leaf tissue (Supplementary Figure 8A) and eigengenes in the blue module were specifically expressed in the leaf tissue (Supplementary Figure 8B).

Hub genes have been reported to be essential for maintaining the structure of the corresponding module and network (Li Y. et al., 2015; van Dam et al., 2018). We found that the blue module contained 22 hub TFs with a module membership value (|kME|) > 0.9, which is designed as the Pearson’s correlation coefficient between the expression of a gene and a given module epigengene (Langfelder and Horvath, 2008). The 22 hub TFs contained 3 TFs enriched with H3K27me3, 4 TFs associated with H3K4me3/H3K27me3 marks, and the rest (15 TFs) enriched with H3K4me3 (Supplementary Figure 9 and Supplementary Table 8).

To further infer interactions between hub TFs, we built a gene regulatory network with 22 hub TFs as candidate regulators for the expression of other genes using the GENIE3 R package (Huynh-Thu et al., 2010; Figure 4A). To clearly show predicted regulatory relationships between TFs, the edges among other genes involved in the network were not displayed. Subsequently, the top 7 TFs with the most edges as displayed in Figure 4A and their regulated genes preferentially expressed in either the A or D subgenome were illustrated in Figure 4B. Strong interactions between TFs occurred among PRE6, BHLH63, RF2b, WOX1, and TCP15. PRE6, BHLH63, and RF2b were regulated by DREB3. To validate the accuracy of the network, we conducted a BiFC assay for Rf2b, bHLH63, and PEE6. Protein interactions were detected between Rf2b/bHLH63 and PEE6, and self-interaction was observed for PRE6 and Rf2b proteins (Figure 4C).

Functions of each TF ortholog have been documented in other plants such as *Arabidopsis* and rice. For example, it has been documented that TCP15 acts as a repressor of auxin biosynthesis and functions in the regulation of *Arabidopsis* gynoecium development (Lucero et al., 2015). DREB3 is a transcriptional activator functioning in abiotic stress responses in plants (Niu et al., 2020). bHLH63 also known as *CRYPTOCHROME-IN-TERACTING* bHLH1 and bHLH100, is mainly involved in embryo suspensor and postembryonic development in *Arabidopsis* (Liu et al., 2008; Radoeva et al., 2019).

Collectively, our analyses indicate that, in addition, to directly affecting the expression of overlapping genes, H3K4me3, H3K27me3, and H3K4me3/H3K37me3 marks can indirectly influence gene expression through TF-mediated regulatory networks in the leaf tissue.

## Discussion

Similar to previous findings in plants (Zhang et al., 2007, 2009; Wang et al., 2009; He et al., 2010; Deal and Henikoff, 2011), our study indicated that H3K4me3 and H3K27me3 are highly enriched in gene bodies, H3K4me3 is an active mark that directly correlates with expression levels of genes, whereas H3K27me3 is a repressive mark that anti-correlates with expression levels of genes in allotetraploid cotton. It has been documented that H3K4me3 is involved in the biased expression of homeologous genes in allotetraploid cotton root (Zheng et al., 2016), and differential enrichment of H3K4me3 is responsible for transcriptional changes of genes associated with cotton development and evolution (You et al., 2017). However, our study for the first time characterized possible roles of H3K27me3, and broad H3K4me3 in differentially regulating gene expression between A and D subgenome in cotton.

### Roles of H3K27me3 and Broad H3K4me3 Enrichment in Gene Transcription in Cotton

Compared with extensive H3K27me3 studies in plant development and stress responses in other plant species (Zheng and Chen, 2011; Gan et al., 2015; Chang et al., 2020), the roles of H3K27me3 in cotton are still much less studied. In addition to the overall repressive role of H3K27me3 in regulating gene expression in cotton, our study showed that distinct enrichment of H3K27me3 in homeologous genes occurred between the A and D subgenomes (Figures 2C–E), since H3K27me3 enrichment in the A subgenome displayed a more pronounced anti-correlation with expression levels of homeologous genes as compared to the D subgenome.

Biological functions of broad H3K4me3 enrichment have been well studied in mammals, namely, cell identity (Benayoun et al., 2014), transcription of tumor suppressor genes (Chen et al., 2015; Dhar et al., 2018), transcription of genes in pre-implantation development, and embryonic stem cell differentiation (Liu et al., 2016), however, it is poorly understood in plants. In addition to the active roles of H3K4me3 in regulating gene expression, our study showed that the effects of H3K4me3 on expression levels of overlapping genes were related to their enrichment levels, relative position, and distance around the TSS in cotton (Figures 3A–C). Importantly, we found that A- and D-specific genes and homeologous genes with broad H3K4me3 in promoters and gene bodies were potentially involved in differential biological relevance and tissue-specific expression, suggesting that broad H3K4me3-marked genes might have some unique biological functions between A and D subgenome. For example, A-specific genes with broad H3K4me3 in promoters had more enriched GO terms relative to those D-specific genes, moreover, they had distinct GO terms associated with molecular functions and biological processes as compared to the corresponding genes with broad H3K4me3 enrichment in gene bodies (Figures 3D,E). It has been reported that genes with broad H3K4me3 enrichment have enriched GO terms associated with photosynthesis in *Arabidopsis* (Brusslan et al., 2015). Thus, our study provides further insights into the roles of broad H3K4me3 enrichment in subfunctionalization of homeologous genes in cotton.

### Impacts of H3K4me3, H3K27me3 and H3K4me3/H3K27me3 Marks on Gene Transcription Through the Regulatory Network

In addition to direct impacts of H3K4me3 and H3K27me3 on transcription of overlapping genes in two subgenomes of cotton, our directional regulatory network related to TF indicated that both marks acted individually or in combined actions to indirectly regulate expression of co-expressed A- or D-biased genes through interacting between H3K4me3 or H3K27me3 mark overlapping TFs. For instance, predicted mutual interactions occurred between BHLH63 and PRE6 or RF2b, and between RF2b and TCP15, some of which were validated using a bimolecular fluorescence complementation assay. TCP 14 and TCP15 can regulate internode length and leaf shape in *Arabidopsis* through modulating cell proliferation (Kieffer et al., 2011). WOX1 is a key regulator during meristem development in *Arabidopsis* (Zhang et al., 2011). RF2b is a bZIP protein functioning in symptom development of rice tungro disease through interacting with RF2a (Dai et al., 2004). Functions of RF2b in leaf and root development in *Arabidopsis* are mediated by bZIP29 (Van Leene et al., 2016). PRE6 is a paclobutrazol resistance protein belonging to non-DNA binding basic helix–loop–helix transcription factor, which has been reported to be involved in phytohormone signaling, such as GA, auxin, and BR and light responses in *Arabidopsis* (Gommers et al., 2017; Zheng et al., 2017). Gene regulatory network has already been applied to predict key nitrogen regulators, followed by successful experimental validation in rice (Ueda et al., 2020). We further extended the gene regulatory network to infer direct or indirect interactions between genes or TFs, which are responsible for differential expression of subgenome specific genes in allotetraploid cotton.

Collectively, our study provides evidence to indicate direct and indirect impacts of H3K4me3 and H3K27me3 on differential transcription of genes or homeologous gene pairs between A and D subgenome in cotton leaf tissue. In particular, the involvement of typically repressive mark H3K27me3 in expression bias of the homoeologs in cotton is still understudied. Therefore, we provide further evidence showing the involvement of H3K27me3 individually or in combination with H3K4me3 in regulating differential expression of homeologous gene pairs between the A and D subgenomes. Thus, H3K27me3 individually or coordinated with H3K4me3 could play important roles in genomic evolution and/or domestication through controlling bias expression of the homoeologs with some specific biological relevance in allotetraploid cotton. It has been reported that methylated genes evolve faster than unmethylated genes, and changes in DNA methylation and H3K4me3 enrichment are directly associated with expression bias of the homoeologs in allotetraploid cotton and their relatives (Song et al., 2017), thereby both epigenetic marks possibly functioning in gene domestication, including flowering time-related gene *GhCOL2* in cotton. Profiling of H3K4me3 and H3K27me3 marks in ancestors and relatives of allotetraploid cotton could help to address epigenetic regulatory roles underlying polyploidization and domestication in cotton.

## Data Availability Statement

The data supporting the findings of this study are available from the corresponding author (WZ, wzhang25@njau.edu.cn), upon request. The raw sequencing data are deposited in the NCBI Gene Expression Omnibus (GEO) (http://www.ncbi.nlm.nih.gov/geo/) under accession number GSE165245.

## Author Contributions

WZ conceived and designed the study and wrote the manuscript with contributions from all the authors. YS, JG, YW, and XD conducted the experiments. AZ analyzed the data. YF, TW, and XC helped with plant growth. RP and ZL helped with the generation of RNA-seq data. FL and KW supervised the experiments. YS, JG, YW, AZ, and WZ interpreted the results. All authors contributed to the article and approved the submitted version.

## Conflict of Interest

The authors declare that the research was conducted in the absence of any commercial or financial relationships that could be construed as a potential conflict of interest.

## Publisher’s Note

All claims expressed in this article are solely those of the authors and do not necessarily represent those of their affiliated organizations, or those of the publisher, the editors and the reviewers. Any product that may be evaluated in this article, or claim that may be made by its manufacturer, is not guaranteed or endorsed by the publisher.

## Footnotes

1
https://cottonfgd.org/

## Supplementary Material

Supplementary Table 1 The homeologous gene pairs between A and D subgenome (in a separate Excel file).

Supplementary Table 2 Primer information was used in this study.

Supplementary Table 3 Summary of sequencing data.

Supplementary Table 4 The expression levels and fold changes of differentially expressed homeologous gene pairs (in a separate Excel file).

Supplementary Table 5 The gene length and expression levels of 5 broad H3K4me3 marked genes from five clusters (in a separate Excel file).

Supplementary Table 6 Details of A- or D-biased TFs and 182 biased TFs with H3K4me3, H3K27me3, or H3K4me3/H3K27me3 marks (in a separate Excel file).

Supplementary Table 7 Summary of A- and D-biased TFs associated with H3K4me3, H3K27me3, and both marks.

Supplementary Table 8 Genes and their kME values in the leaf-related blue module (in a separate Excel file).
