# Supplementary material for: Profiling of H3K4me3 and H3K27me3 and Their Roles in Gene Subfunctionalization in Allotetraploid Cotton
Source: Front Plant Sci. 2021 Dec 15;12:761059. doi: 10.3389/fpls.2021.761059 (PMC8714964; doi:10.3389/fpls.2021.761059)
Supplement: Supplementary Table 2 — Primer information was used in this study. [file Data_Sheet_1.PDF]

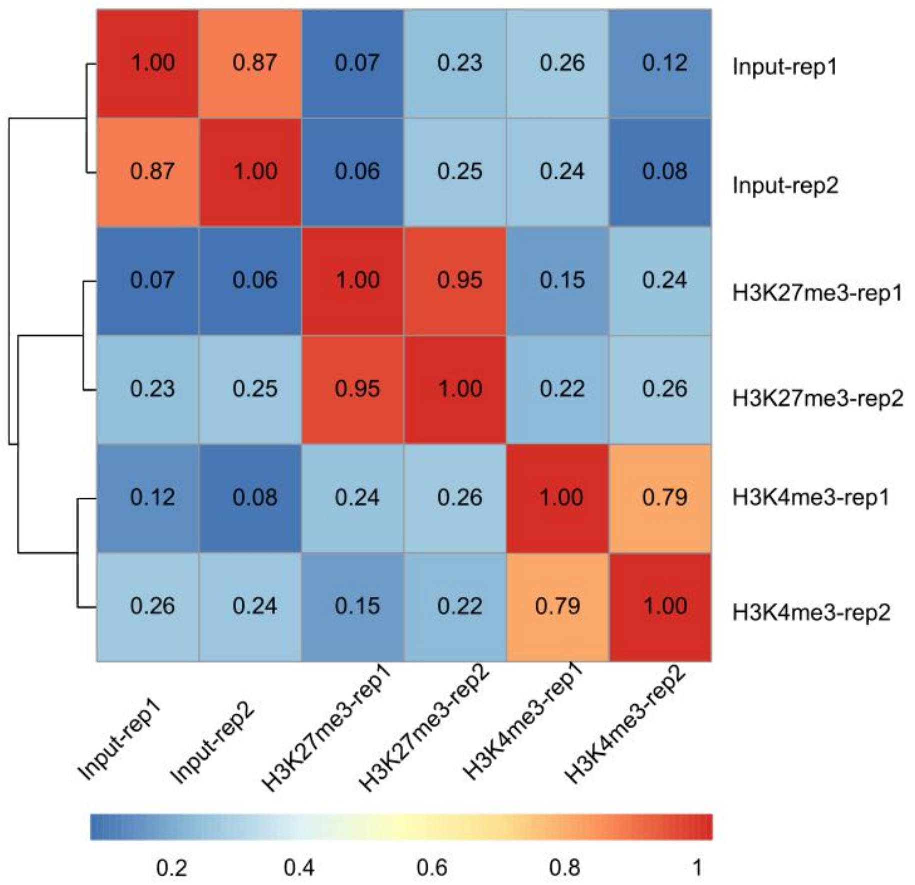

Supplementary Fig. S1. Correlation analyses of biologically replicated input, H3K27me3 and H3K4me3 ChIP-seq reads. Spearman's rank correlation coefficient (r) is shown**.**

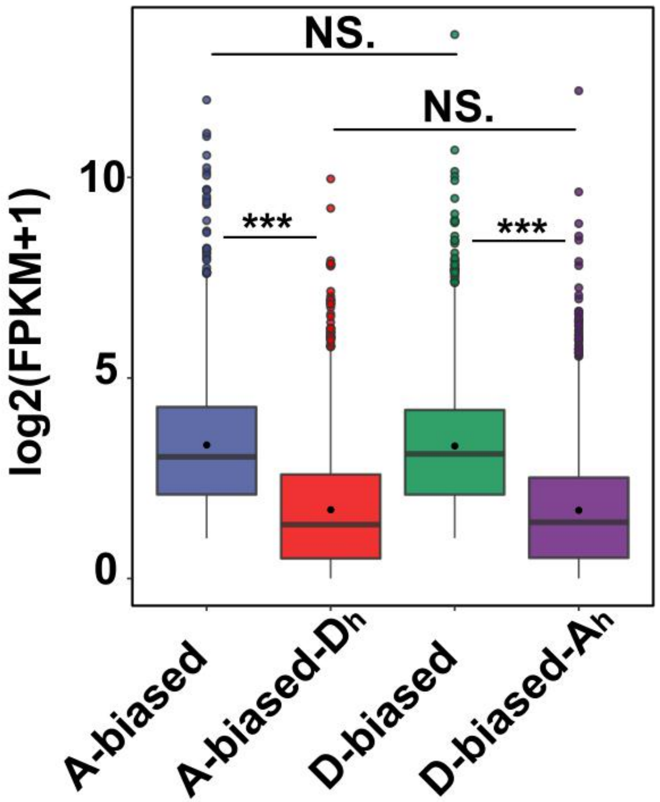

Supplementary Fig. S2. Expression levels of biased genes and their corresponding homeologous genes. Significance test was determined using Wilcoxon rank sum test, ***p < 0.001.

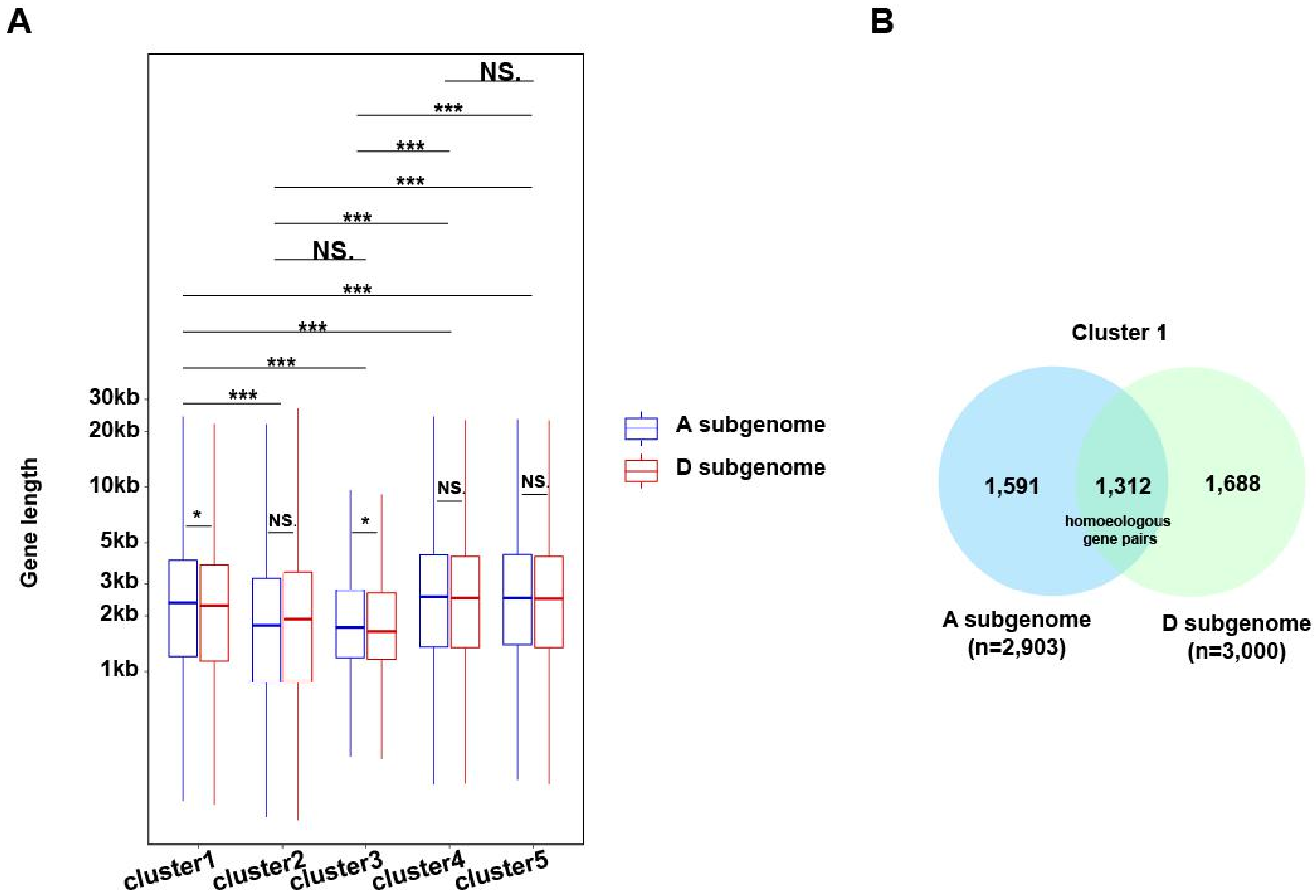

Supplementary Fig. S3. Characterization of the genes in each cluster. (**A**) Gene length of H3K4me3 overlapping genes in five clusters from two subgenomes. (**B**) Venn plots illustrating A subgenome-only, D subgenome-only and homeologous gene pairs in Cluster 1.

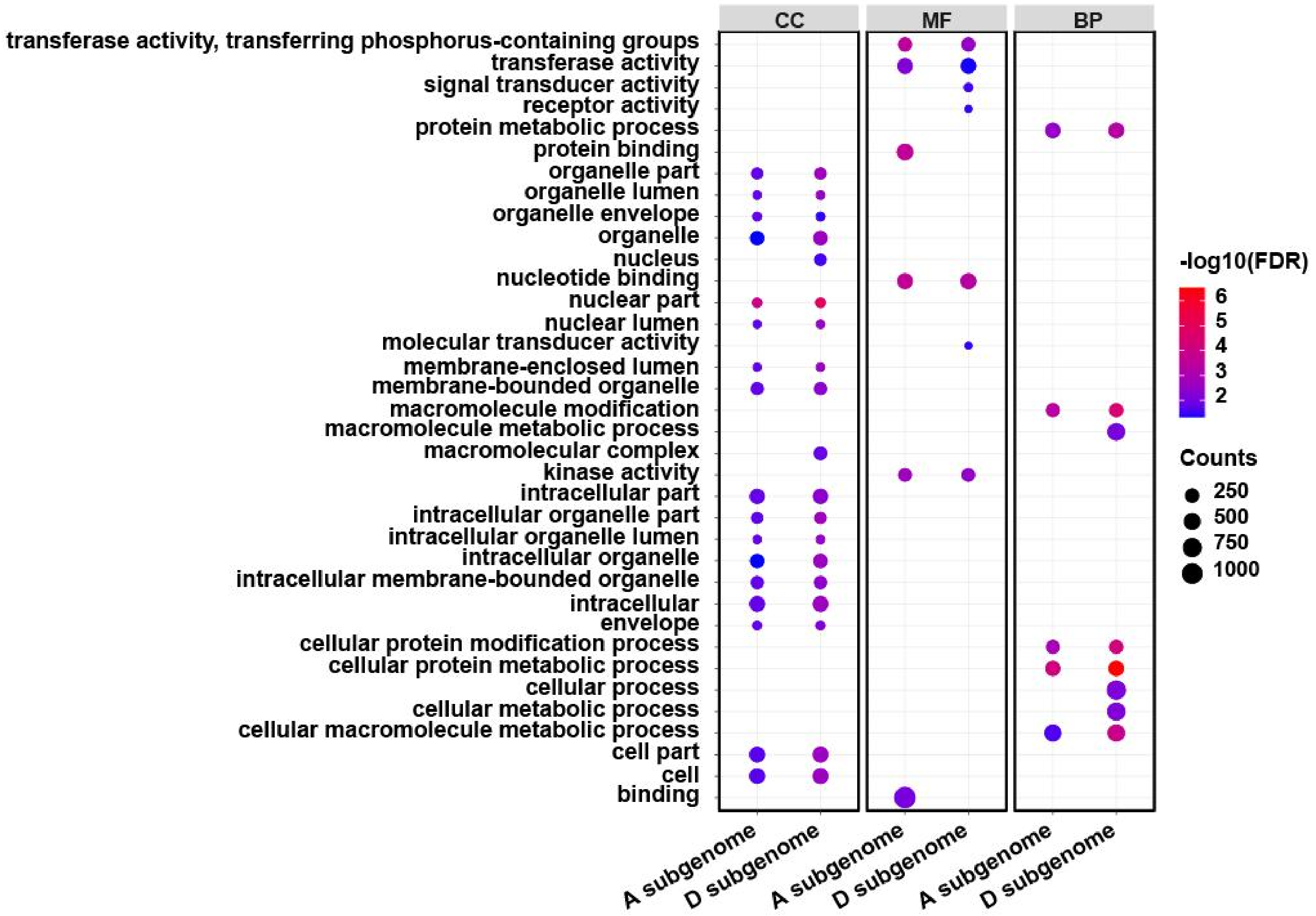

Supplementary Fig. S4. Functional GO term enrichment analyses of the genes from Cluster1. The size of each dot represents the number of genes, and the color key indicates -log10(FDR).

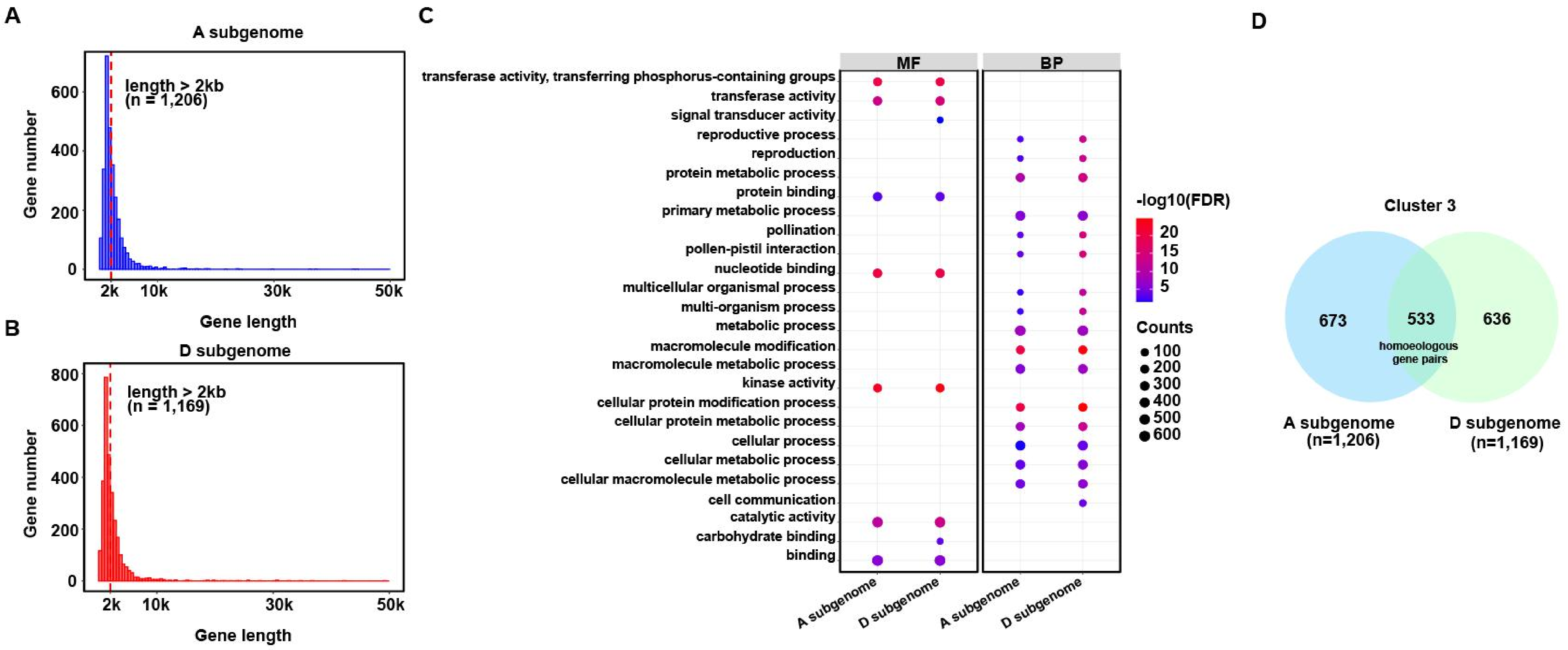

Supplementary Fig. S5. Characterization of the genes in Cluster 3. The number of genes with different length from A subgenome (**A**) and D subgenome (**B**) in Cluster 3, genes with length > 2 kb were noted. (**C**) Functional GO term enrichment analyses of genes from Cluster3, the size of each dot represents the number of genes, and the color key indicates -log10(FDR). (**D**) Venn plots illustrating the number of genes in A subgenome-only , D subgenome-only and homeologous gene pairs in Cluter 3.

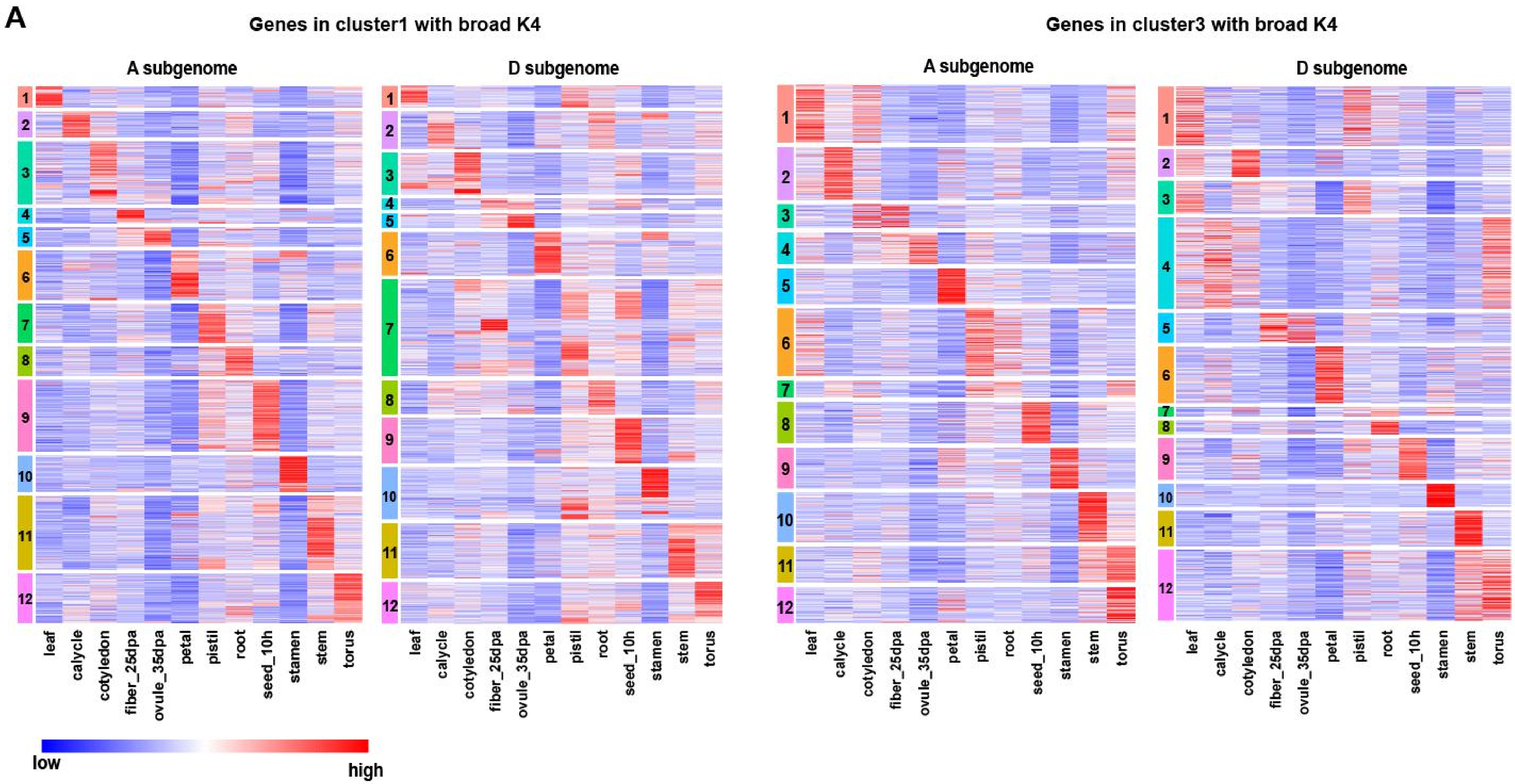
A
Genes in cluster1 with broad K4
Genes in cluster3 with broad K4
A subgenome
D subgenome
A subgenome
D subgenome
leaf
calycle
cotyledon
fiber_25dpa
ovule_35dpa
petal
pistil
root
seed_10h
stamen
stem
torus
1
2
3
4
5
6
7
8
9
10
11
12
low
high

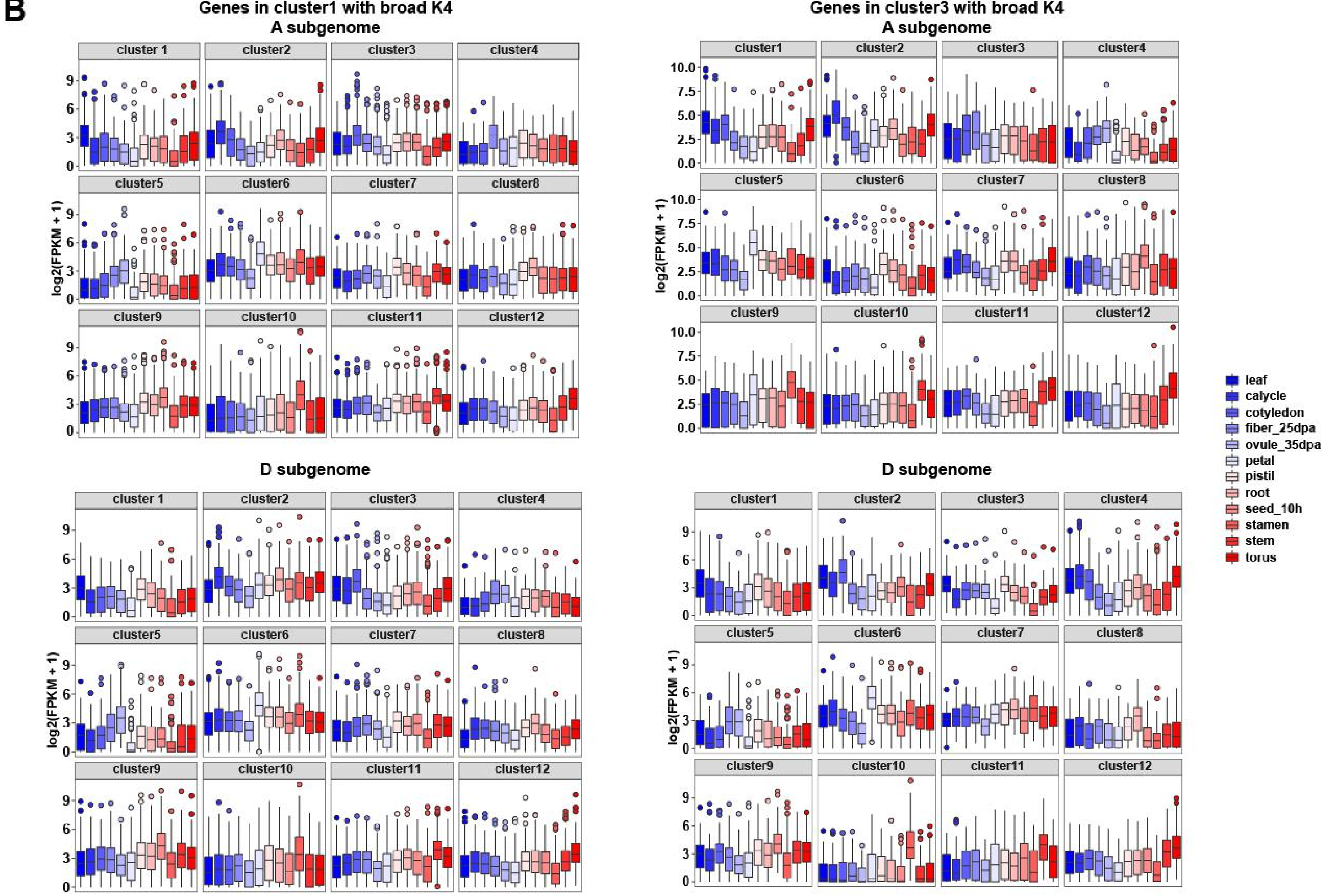

Supplementary Fig. S6. Tissue specific expression of the genes in Cluster 1 and Cluster 3. (A) Heatmaps showing expression profiles of the genes in Cluster 1 and Cluster 3 across twelve different tissues from A subgenome and D subgenome, respectively, the color key indicates the z-score. (**B**) Expression levels of genes in A subgenome and D subgenome in Cluster 1 and Cluster 3, the clusters correspond to the ones characterized in Figure S6A.

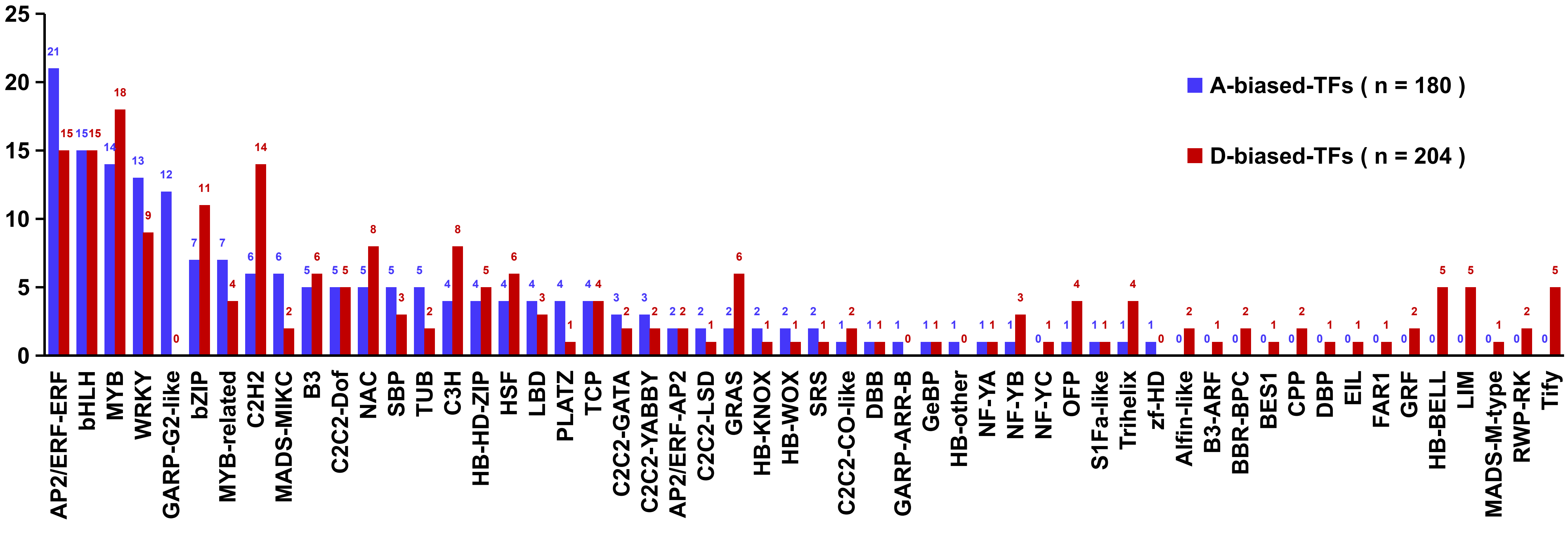

Supplementary Fig. S7. The number of A-biased and D-biased TFs in cotton leaf tissue.

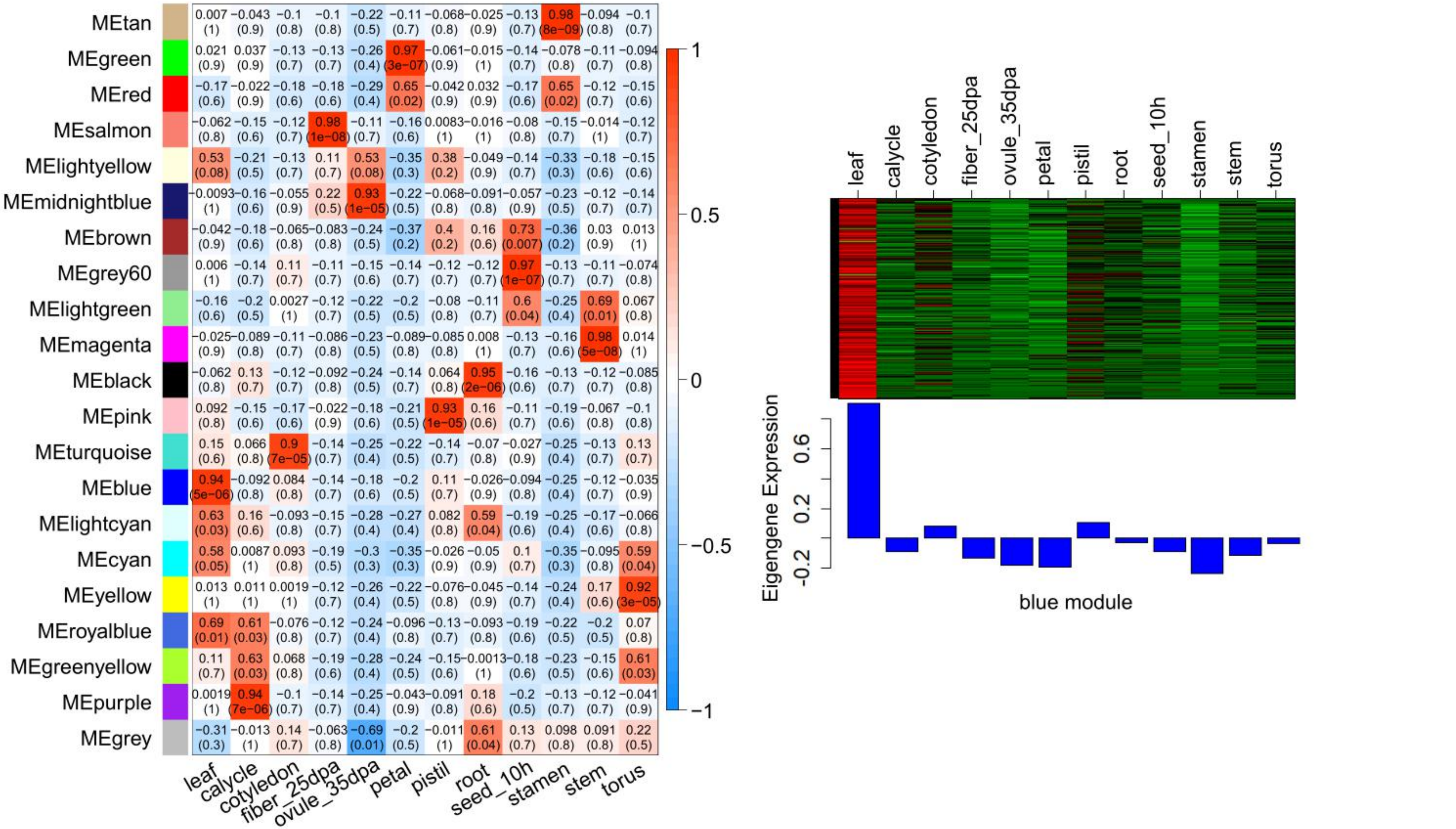

Supplementary Fig. S8 Gene co-expression assay. (**A**) The heatmap showing a correlation coefficient between the module and sample, the color key represents a degree of correlation between a specific module and a sample. (**B**) Expression pattern of genes in the blue module, which contains genes specifically expressed in the leaf tissue.

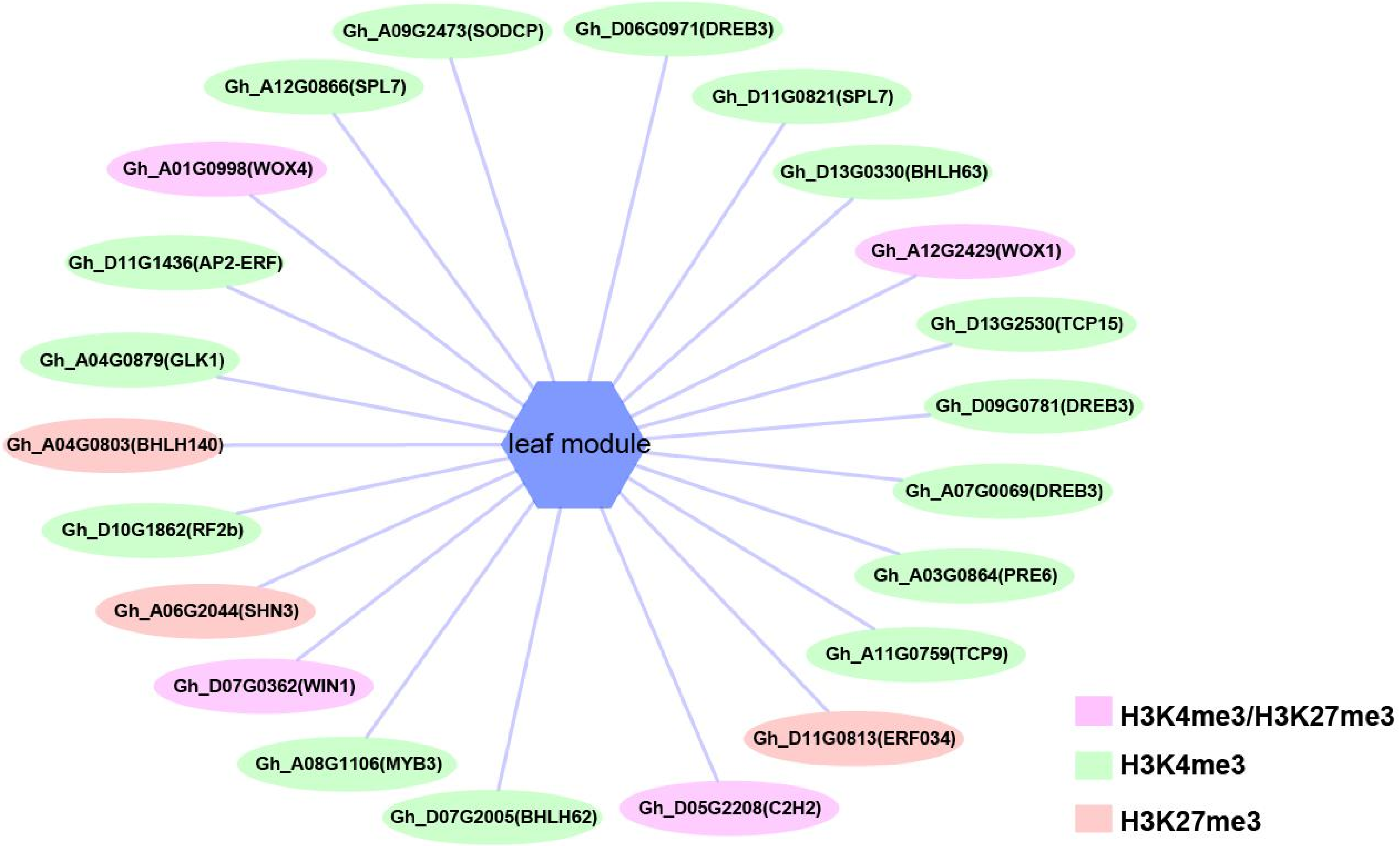

Supplementary Fig. S9. Illustration of hub TFs with different marks derived from the blue module. Pearson's correlation coefficient between genes vs module eigengene expression was calculated, which is defined as 'kME'. Genes with |kME| > 0.9 were defined as hub genes.

Supplementary Table S2. Primer information used in this study

Primer names	Primer sequences (5'-3')
Rf2b-106 F	atcgaggacgccggcggatccATGCAGGATCCACCAAGTCTG
Rf2b-106 R	caggtcgactctagaggatccTCATAATGTTCCACTACTTTC
bHLH63-106 F	atcgaggacgccggcggatccATGAACACAGCATTGCCGGA
bHLH63-106 R	caggtcgactctagaggatccTCATATCTCCTTCAGATCAC
PRE6-106 F	atcgaggacgccggcggatccATGTCAGGCAGAAGATCACG
PRE6-106 R	caggtcgactctagaggatccTTATTGCATAAGTAAACTCCT
RF2b-104 F	attacaggtacccggggatccATGCAGGATCCACCAAGTCTG
RF2b-104 R	gccgtcgactctagaggatccTAATGTTCCACTACTTTCAC
bHLH63-104 F	attacaggtacccggggatccATGAACACAGCATTGCCGGA
bHLH63-104 R	gccgtcgactctagaggatccTATCTCCTTCAGATCACTTG
PRE6-104 F	attacaggtacccggggatccATGTCAGGCAGAAGATCACG
PRE6-104 R	gccgtcgactctagaggatccTTGCATAAGTAAACTCCTTA
RF2b-62SK F	atcccccgggctgcaggaattcATGCAGGATCCACCAAGTCTG
RF2b-62SK R	cgataagcttgatatcgaattcTCATAATGTTCCACTACTTTC
PRf2b-0800 F	ggtcgacggtatcgataagcttGATAGTGTAATATTTAGGCA
PRf2b-0800 R	gcaggaattcgatatcaagcttGTGGTAAGAGCCTCGGAAAG
bHLH63-62SK F	atcccccgggctgcaggaattcATGAACACAGCATTGCCGGA
bHLH63-62SK R	cgataagcttgatatcgaattcTCATATCTCCTTCAGATCAC
PbHLH63-0800 F	ggtcgacggtatcgataagcttCATTGTTAATACAATCGATTC
PbHLH63-0800 R	gcaggaattcgatatcaagcttCAAGCGATCCCTTTGCCTCTC
PRE6-62SK F	atcccccgggctgcaggaattcATGTCAGGCAGAAGATCACG
PRE6-62SK R	cgataagcttgatatcgaattcTTATTGCATAAGTAAACTCCT
PPRE6-0800 F	ggtcgacggtatcgataagcttTCATTGTACCTTAATTAACTC
PPRE6-0800 R	gcaggaattcgatatcaagcttAGGGTTCGAGTCTACTCCGAA

Supplementary Table S3. Summary of sequencing data

Samples	No. of Clean reads	Mapped reads	Mapping ratio	Uniquely mapped reads (MapQ >30)	Uniquely mapped ratio
Leaf-RNA-seq-rep1	72,336,886	69,330,326	95.84%	56,304,020	77.83%
Leaf-RNA-seq-rep2	69,698,414	66,666,709	95.65%	53,882,563	77.31%
Leaf-RNA-seq-rep3	73,945,114	69,972,875	94.63%	55,724,386	75.36%
Input-rep1	22,714,740	22,418,221	98.69%	11,732,686	51.65%
Input-rep2	34,557,734	33,887,901	98.06%	17,401,383	50.35%
H3K4me3-rep1	29,175,244	28,852,803	98.89%	16,200,284	55.53%
H3K4me3-rep2	33,142,614	32,789,553	98.93%	18,021,828	54.38%
H3K27me3-rep1	51,547,766	50,629,738	98.22%	31,389,930	60.89%
H3K27me3-rep2	49,151,156	47,019,719	95.66%	25,366,108	51.61%

Supplementary Table S7. Summary of A- and D-biased TFs associated with H3K4me3, H3K27me3 and both marks

TF	A-biased				D-biased				TF	A-biased				D-biased			
	K4	K4/K27	K27	No.	K4	K4/K27	K27	No.		K4	K4/K27	K27	No.	K4	K4/K27	K27	No.
AP2/ERF-ERF	18	1	1	20	10	2	1	13	C2C2-CO-like	1	0	0	1	2	0	0	2
bHLH	8	4	2	14	13	1	0	14	DBB	1	0	0	1	1	0	0	1
MYB	10	4	0	14	9	6	1	16	GARP-ARR-B	1	0	0	1	0	0	0	0
WRKY	12	2	0	14	7	1	0	8	GeBP	1	0	0	1	1	0	0	1
GARP-G2-like	9	2	0	11	0	0	0	0	HB-other	0	0	0	0	0	0	0	0
bZIP	4	1	0	5	8	1	0	9	NF-YA	1	0	0	1	0	0	0	0
MYB-related	5	0	0	5	1	0	1	2	NF-YB	0	0	0	0	2	1	0	3
C2H2	5	0	0	5	6	5	0	11	NF-YC	0	0	0	0	1	0	0	1
MADS-MIKC	0	3	3	6	0	1	1	2	OFP	1	0	0	1	1	3	0	4
B3	3	2	0	5	5	0	0	5	S1Fa-like	1	0	0	1	1	0	0	1
C2C2-Dof	1	1	2	4	3	2	0	5	Trihelix	1	0	0	1	3	1	0	4
NAC	2	1	0	3	8	0	0	8	zf-HD	1	0	0	1	0	0	0	0
SBP	5	0	0	5	2	0	0	2	Alfin-like	0	0	0	0	1	0	0	1
TUB	5	0	0	5	2	0	0	2	B3-ARF	0	0	0	0	1	0	0	1
C3H	3	0	0	3	6	0	0	6	BBR-BPC	0	0	0	0	2	0	0	2
HB-HD-ZIP	2	1	1	4	4	0	0	4	BES1	0	0	0	0	1	0	0	1
HSF	3	1	0	4	6	0	0	6	CPP	0	0	0	0	1	0	0	1
LBD	2	1	1	4	0	3	0	3	DBP	0	0	0	0	1	0	0	1
PLATZ	4	0	0	4	0	1	0	1	EIL	0	0	0	0	1	0	0	1
TCP	4	0	0	4	3	1	0	4	FAR1	0	0	0	0	1	0	0	1
C2C2-GATA	2	1	0	3	1	1	0	2	GRF	0	0	0	0	1	1	0	2
C2C2-YABBY	0	2	1	3	0	1	1	2	HB-BELL	0	0	0	0	4	1	0	5
AP2/ERF-AP2	2	0	0	2	1	1	0	2	LIM	0	0	0	0	5	0	0	5
C2C2-LSD	2	0	0	2	0	0	0	0	MADS-M-type	0	0	0	0	0	0	0	0
GRAS	0	1	0	1	5	1	0	6	RWP-RK	0	0	0	0	2	0	0	2
HB-KNOX	2	0	0	2	1	0	0	1	Tify	0	0	0	0	3	2	0	5
HB-WOX	0	2	0	2	1	0	0	1									
SRS	1	1	0	2	0	1	0	1	Sum.	123	31	11	165	139	38	5	182
