# Supplementary figures and images for: Profiling of H3K4me3 and H3K27me3 and Their Roles in Gene Subfunctionalization in Allotetraploid Cotton
Source: Front Plant Sci. 2021 Dec 15;12:761059. doi: 10.3389/fpls.2021.761059 (PMC8714964; doi:10.3389/fpls.2021.761059)

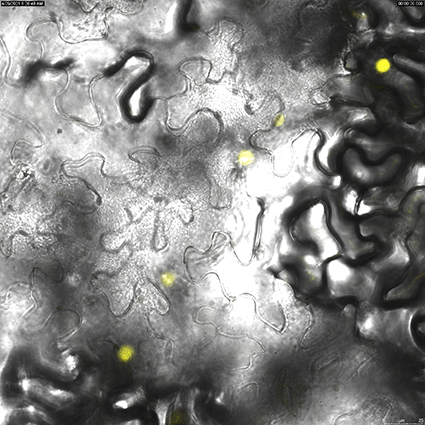

Supplement: Supplementary file 7 [file Image_1.TIF]

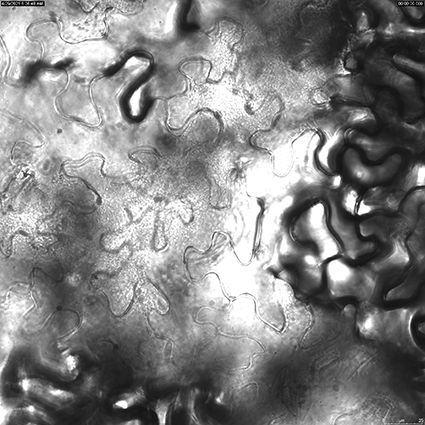

Supplement: Supplementary file 8 [file Image_2.TIF]

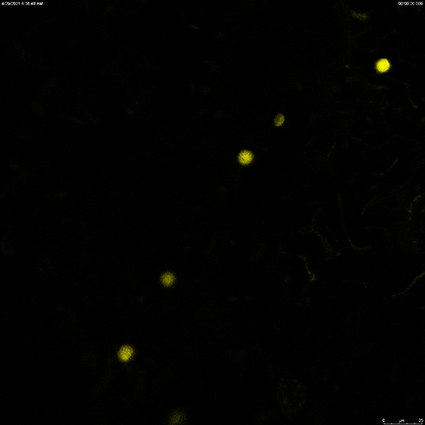

Supplement: Supplementary file 9 [file Image_3.TIF]

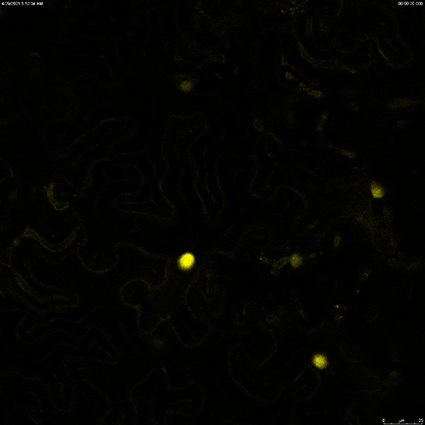

Supplement: Supplementary file 10 [file Image_4.TIF]

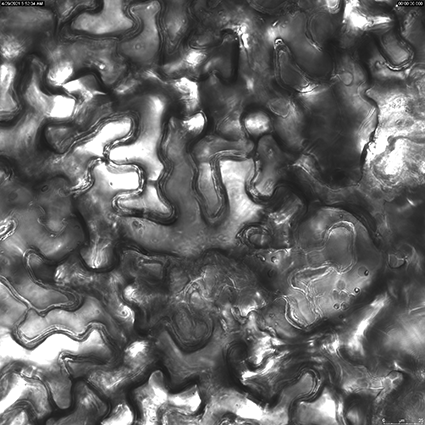

Supplement: Supplementary file 11 [file Image_5.TIF]

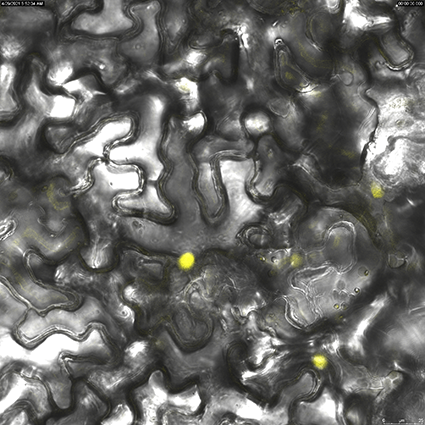

Supplement: Supplementary file 12 [file Image_6.TIF]

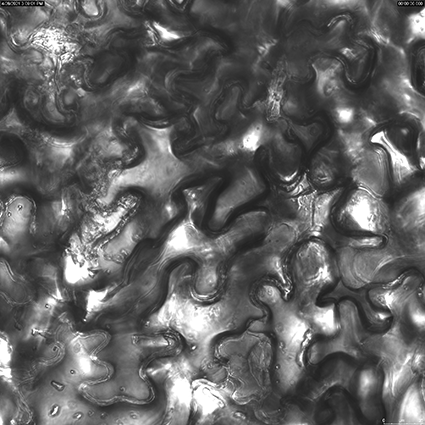

Supplement: Supplementary file 13 [file Image_7.TIF]

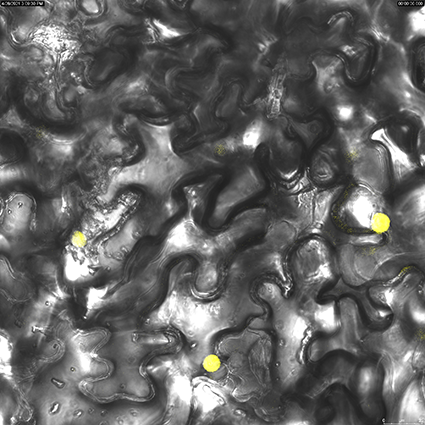

Supplement: Supplementary file 14 [file Image_8.TIF]

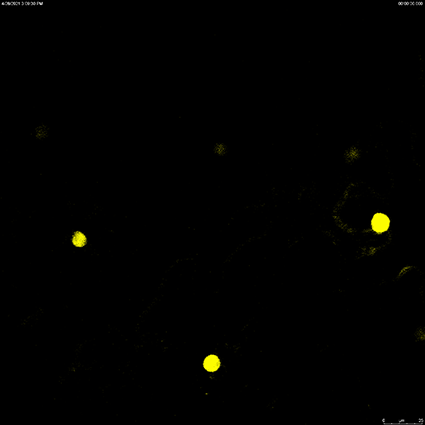

Supplement: Supplementary file 15 [file Image_9.TIF]

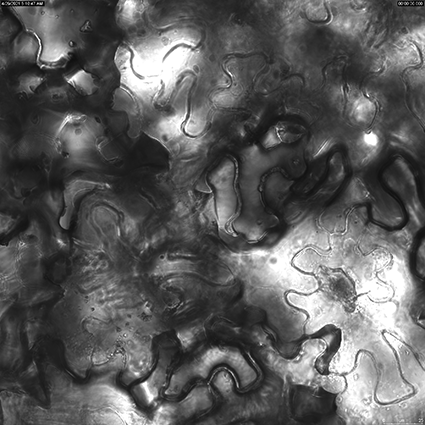

Supplement: Supplementary file 16 [file Image_10.TIF]

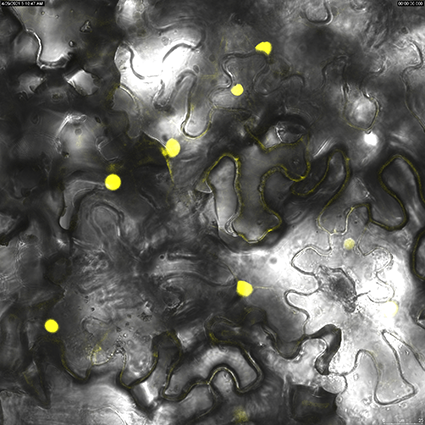

Supplement: Supplementary file 17 [file Image_11.TIF]

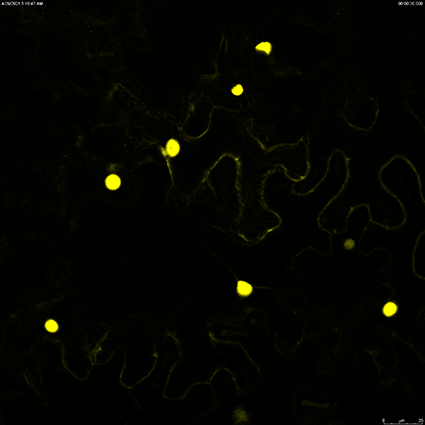

Supplement: Supplementary file 18 [file Image_12.TIF]
